# An in-memory computing architecture based on two-dimensional semiconductors for multiply-accumulate operations

**DOI:** 10.1038/s41467-021-23719-3

**Published:** 2021-06-07

**Authors:** Yin Wang, Hongwei Tang, Yufeng Xie, Xinyu Chen, Shunli Ma, Zhengzong Sun, Qingqing Sun, Lin Chen, Hao Zhu, Jing Wan, Zihan Xu, David Wei Zhang, Peng Zhou, Wenzhong Bao

**Affiliations:** 1 State Key Laboratory of ASIC and System, School of Microelectronics, Fudan University, Shanghai, China; 2 Shenzhen Sixcarbon Technology, Shenzhen, China

**Keywords:** Electronic devices, Two-dimensional materials, Computational nanotechnology

## Abstract

In-memory computing may enable multiply-accumulate (MAC) operations, which are the primary calculations used in artificial intelligence (AI). Performing MAC operations with high capacity in a small area with high energy efficiency remains a challenge. In this work, we propose a circuit architecture that integrates monolayer MoS_2_ transistors in a two-transistor–one-capacitor (2T-1C) configuration. In this structure, the memory portion is similar to a 1T-1C Dynamic Random Access Memory (DRAM) so that theoretically the cycling endurance and erase/write speed inherit the merits of DRAM. Besides, the ultralow leakage current of the MoS_2_ transistor enables the storage of multi-level voltages on the capacitor with a long retention time. The electrical characteristics of a single MoS_2_ transistor also allow analog computation by multiplying the drain voltage by the stored voltage on the capacitor. The sum-of-product is then obtained by converging the currents from multiple 2T-1C units. Based on our experiment results, a neural network is ex-situ trained for image recognition with 90.3% accuracy. In the future, such 2T-1C units can potentially be integrated into three-dimensional (3D) circuits with dense logic and memory layers for low power in-situ training of neural networks in hardware.

## Abstract (web summary)

In standard computing architectures, memory and logic circuits are separated, a feature that slows matrix operations vital to deep learning algorithms. Here, the authors present an alternate in-memory architecture and demonstrate a feasible approach for analog matrix multiplication.

## Introduction

Artificial intelligence (AI) algorithms require significant computing power for running successive matrix calculations. Multiply accumulate (MAC) is the most critical operation in AI computation at the chip level. In-memory computing is a technology that uses memory devices assembled in an array to execute MAC operations^1^. As such, it has triggered extensive research interests because data transfer in a conventional von Neumann architecture has a bottleneck between memory and logic circuits^2,3^, and a memory device capable of in-memory computing can be used to carry out high-throughput MAC operations directly^4,5^. For an ideal in-memory computing, various features are preferred for its memory portion, including a nonvolatile characteristic, multi-bit storage capability, long cycling endurance, simple erase/write operation, etc.^1,4,6^.

Various types of memory devices have been investigated for performing MAC operations. Among them, nonvolatile memory devices, include resistive random-access memory (RRAM)^7–9^, phase change RAM (PCRAM)^10–13^, spin-transfer torque magnetoresistive RAM (STT-MRAM)^14,15^, and conventional FLASH^16–18^. Most nonvolatile memories can realize multi-bit storage, but they usually exhibit a stochastic nature, resulting in a learning accuracy loss in the neural network applications^1,5^. Their limited cycling endurance (FLASH ~10^5^, RRAM and PCRAM 10^6^–10^9^) and relatively complex memory operation^19^ are also unsuitable for frequent weight update processes required for in-memory computing^20^. For example, FLASH usually requires high voltages for the write operation. RRAM/PCRAM requires continuous voltage pulses to tune the conductive filaments to control the electrical conductance, which complicates the multiplication operation^5^. STT-MRAM requires a relatively large current to program information in the storage element, which carries greater dynamic power dissipation and overall write energy cost^4,15^. On the other hand, volatile memory devices can also execute in-memory computing, such as static random accesses memory (SRAM)^21–23^ and dynamic random-access memory (DRAM)^24–26^. Theoretically, they have much higher programming speed and superior endurance(>10^16^)^1,4^, but in volatile memories, the stored information dissipates quickly, and a periodic refresh operation is required^24^. Furthermore, SRAM and DRAM belong to binary memory, and their main applications are limited in the binary-weighted network^1,25,26^. An overall comparison among different types of in-memory computing technologies is also concluded in Supplementary Table S1.

Other than exploring different memory technologies for in-memory computation, suitable channel material is also critical. Two-dimensional layered materials (2DLMs), well-known for their intrinsic nature of atomic thickness, allow aggressive channel length scaling owing to its superior electrostatic control that can substantially suppress short-channel effects^27^. In addition, unlike rigid silicon CMOS, 2DLMs can enable flexible electronic circuitry with multiple sensing functionalities, adding value towards a multifunctional hardware platform^28^. Among various 2DLMs, semiconductive transition metal dichalcogenides (TMDs) are promising due to their rich band structures and tunable bandgaps^29^, and molybdenum disulfide (MoS_2_) is one representative that has been extensively investigated in the past few years^30,31^. Compared to silicon and other TMDs with a narrower bandgap, monolayer MoS_2_ has a relatively wide bandgap (~1.8 eV) to enable a large current on/off ratio in its field-effect transistors (FETs)^32^. Now wafer-scale continuous MoS_2_ films can already be synthesized by chemical vapor deposition (CVD) methods^33^ and transferred to arbitrary substrates^34^. The device processing techniques have also been intensively investigated to address early criticism of 2D-FETs, such as the realization of Ohmic contact and integration of high-k dielectrics^35–37^. Therefore, recent exploration of 2DLMs has been expanded from fundamental investigations to the demonstration of circuit-level device applications, such as memories, logic gates, and sensors^35,38,39^. A 1T-1R structured in-memory computation unit has also been demonstrated lately, in which a MoS_2_ FET is used as a selector, and a HfOx-based RRAM is used to perform analog calculation^40^.

In this work, we explored and designed a MAC circuit architecture in a 2T–1C configuration, which includes two MoS_2_ FETs and one metal-insulator-metal capacitor. In such a structure, the 1T–1C portion acts as a DRAM cell. Owing to the ultralow leakage current of the MoS_2_ FETs, a voltage with 8-level (3 bits) quantization can be stored on a capacitor with longer than 10 s retention time, enough for additional complex operations. The stored voltage is connected to the gate of the second MoS_2_ transistor, in which the input drain bias *V*_*d*_ and gate bias *V*_*g*_ can determine the drain current *I*_*d*_ to realize an analog multiplication operation. Moreover, the current in multiple 2T–1C rows can be converged together, giving an addition operation. Based on two identical 2T–1C cells, we demonstrate a simple MAC operation circuit, which is the core module for the convolution operation in an artificial neural network. A more complicated MAC array was trained against the MNIST handwritten digit database and used for image recognition. The successful recognition rate was found to reach 90.3%. Our 2T–1C MoS_2_ cells highlight the promising potential of in-memory computing and in situ training of neural networks based on emerging 2D semiconductors to overcome the bottleneck of von Neumann computing.

## Results and discussion

**Fig. 1:**
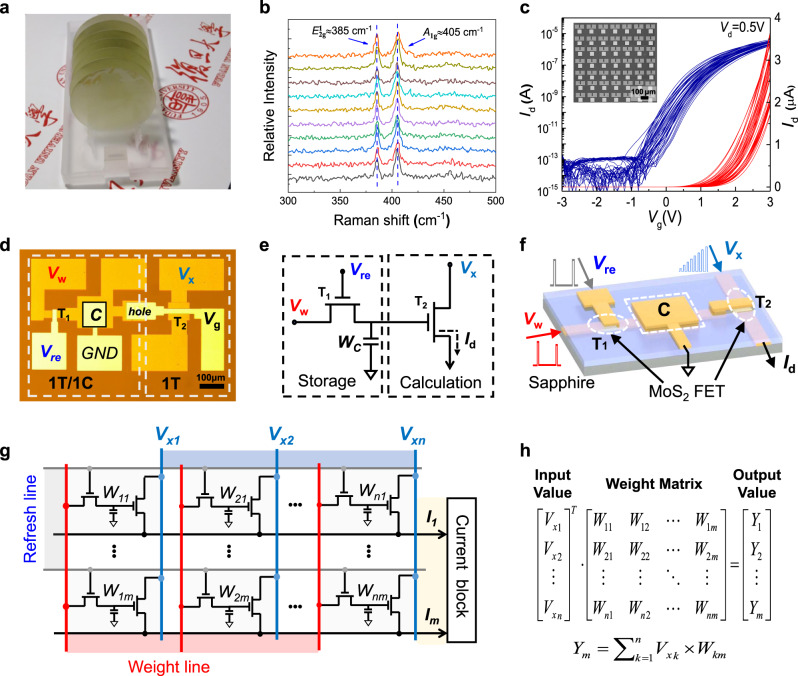
2T–1C unit cell and circuits fabricated on a wafer-scale MoS_2_ film. **a** Wafer-scale MoS_2_ continuous films are batch-synthesized by a CVD method. **b** Raman spectra from different positions on the MoS_2_ film. **c** Transfer characteristics for 24 MoS_2_ transistors spread on a 2 in. wafer. **d** Microscope image of the fabricated 2T–1C cell. Scale bar: 100 μm. **e** Circuit schematic of a 2T–1C cell containing storage and calculation modules. **f** 3D schematic illustration of a 2T–1C unit cell, including two MoS_2_ FETs and one capacitor. **g** Circuit diagram of the proposed 2T–1C cell array. **h** A typical diagram of a matrix convolution operation.

Figure 1a shows a wafer-scale MoS_2_ film grown using the CVD method (see SI). Raman spectra (Fig. 1b) gathered from different positions in the MoS_2_ film show acceptable spatial uniformity, which is vital for performing accurate analog calculations in our circuit. The transfer characteristics (Fig. 1c) of 24 MoS_2_ FETs on a 1 × 1 cm^2^ wafer exhibit large on/off current ratios (~10^7^) and an acceptable homogeneity level. We fabricated a 2T–1C cell (optical microscopic image shown in Fig. 1d) to provide charge storage and analog computation. Figure 1e shows a circuit schematic of such a 2T–1C cell; the left 1T–1C structure forms a dynamic memory in which the MoS_2_ FET is labeled T_1_, and the MoS_2_ FET T_2_ on the right side is used to accomplish the multiplication calculation. Figure 1f schematically illustrates its 3D structure, and the fabrication process is described in the “Methods” section.

The refresh voltage *V*_re_ on the refresh line (RL) controls the ON/OFF state of transistor T_1_. During a write operation, T_1_ is turned on and the signal *V*_*w*_ applied by the weight line (WL) then charges the capacitor, which indicates the weight has been written into this 2T–1C cell. During the hold operation, T_1_ is turned off by applying a negative *V*_re_. Due to the ultralow leakage current in the MoS_2_ channel in the OFF state (see Fig. S1), the charge stored in the capacitor can be held for a long time to maintain the voltage that acts as a gate voltage for T_2_. Since the input *V*_*x*_ is applied as a drain voltage to T_2_, the drain current (*I*_*d*_) in T_2_ is controlled with a combination of *V*_*w*_ and *V*_*x*_. If the applied *V*_*w*_ and *V*_*x*_ locate in a relatively linear range of the output and transfer characteristics for the MoS_2_ FET, an analog multiplication operation between *I*_*d*_, *V*_*x*_, and *V*_*w*_ can be realized, which will be discussed in detail later in this paper.

We now propose an array circuit based on such a MoS_2_ 2T–1C unit cell to implement a MAC operation in an electrical circuit. The circuit diagram is displayed in Fig. 1g, which corresponds to a MAC operation ${Y}_{m}=\mathop{\sum }\limits_{k=1}^{n}{V}_{{\rm{x}}k}\times {W}_{{km}}$ (Fig. 1h). In each unit, the weight *W*_*nm*_ is stored in the capacitor and updated using the RL and WL. The input voltage *V*_*xn*_ is then applied to the entire column *n*. Both *W*_*nm*_ and *V*_*x*_ determine the drain current *I*_*d*_ in each MoS_2_ FET. Finally, the output currents in all rows are added to give a total current *I*_*m*_. The collected current then flows into the current block for further calculation. The relationship between ${I_{m}},\,{W_{nm}},\,{\mathrm{and}}\,{V_{x}}\,{\mathrm{is}}\,{I}_{m}=\mathop{\sum }\limits_{k=1}^{n}{\rm{g}}\,(V_{{\rm{x}}k},{W}_{{km}})$, where ${\rm{g}}\left(x\right)$ is a current–voltage transform function that depends on the transfer and output characteristics of transistor T_2_. Below we will try to build a correlation between *Y*_*m*_ and *I*_*m*_.

**Fig. 2:**
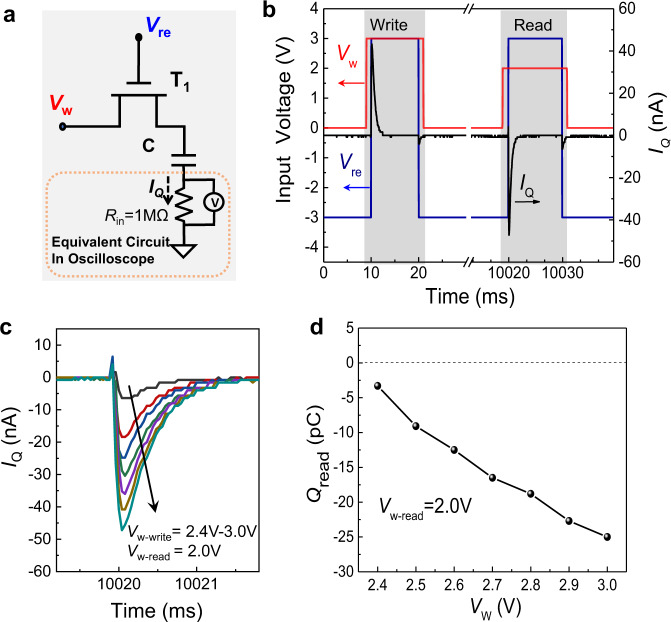
Characterization of the 1T–1C storage module. **a** Schematic diagram of the electrical circuit used to measure the 1T–1C storage module (shadow area). The equivalent circuit in the dashed box equals an external oscilloscope connected to the capacitor. **b** Input voltage waveform (*V*_*w*_, *V*_re_) and readout current (*I*_*Q*_) vs. measurement time. **c** *I*_*Q*_ spikes at *V*_re_ = 3 V while *V*_*w*−write_ ranges from 2.4 to 3 V in 0.1 V steps. **d** Calculated retained charge (*Q*_read_) in the capacitor as a function of *V*_*w*−write_ (compared with *V*_*w*−read_ = 2.0 V when *I*_*Q*_ = 0 A).

We first characterize the properties of the 1T–1C storage module. Figure 2a shows a schematic diagram of the measurement circuit, in which one end of the capacitor is connected to an external oscilloscope (see Fig. S4 for more details). The internal resistance of the oscilloscope *R*_in_ is used to estimate the current flow (*I*_*Q*_) during read/write operations by measuring the voltage of *R*_in_. To measure *I*_*Q*_, voltage signals *V*_*w*_ and *V*_re_ are applied to T_1_ (Fig. 2b) with pulse widths of 12 and 10 ms, respectively. *V*_*w*_ rises 1 ms earlier than *V*_re_ and falls 1 ms later than *V*_re_ to ensure the charge is entirely written onto the capacitor and prevent leakage current through T_1_. *V*_re_ and *V*_*w*_ were both set to 3 V during the write operation. The high *V*_re_ value turns on T_1_, allowing *V*_*w*_ to charge the capacitor to the same potential. A positive current pulse (*I*_*Q*_^+^) during the write operation indicates a charge flows into the capacitor. After the write operation completes, *V*_re_ is switched to −3 V to turn off T_1_. Due to the ultra-low leakage current (Fig. S1), the charged voltage on the capacitor can be stably maintained during the write operation. After waiting for 10 s, a read operation is triggered, where *V*_re_ = 3 V and *V*_*w*−read_ = 2 V. The polarity of the measured *I*_*Q*_ pulse is now negative, indicating the capacitor potential is higher than 2 V and charge flows out of the capacitor. In contrast, if the capacitor potential is less than 2 V, the capacitor will be recharged again, giving a positive current pulse. To further characterize the dependence of *V*_*w*−write_ for reading *I*_*Q*_, the above measurements were repeated. Figure 2c shows the *I*_*Q*_ pulses for reading under various values of writing *V*_*w*-wirte_. To estimate the charge in the capacitor, after waiting for 10 s, *V*_*w*−read_ = 2 V is applied to compare with the retained capacitor voltage to read the remaining charge. The amplitude of the *I*_*Q*_ pulse becomes larger as *V*_*w*_ increases. It is also noted that all *I*_*Q*_ pulses are under 2 ms (Fig. 2c), which approximately equals the write time. The integral of the current overtime during a read cycle equals the charge *Q*_read_ remaining after the waiting interval (10 s). In Fig. 2d, the calculated *Q*_read_ vs. *V*_*w*_ curve is linear, indicating that the charge saved on the capacitor can still be differentiated after 10 s.

**Fig. 3:**
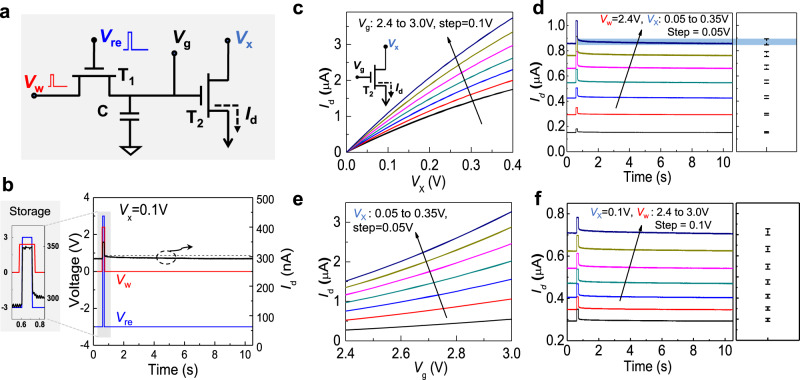
Characterization of the 2T–1C unit cell. **a** Schematic diagram of the circuit used to gather measurements from a 2T–1C unit cell. **b** A complete storage and calculation operation for a 2T–1C unit. The input voltage (*V*_*w*_, *V*_re_, *V*_*x*_) and drain current (*I*_*d*_) are shown vs. measurement time for a 10 s cycle. The magnified inset shows details of the refresh operation. **c** The output characteristics for T_2_ with *V*_*g*_ ranging from 2.4 to 3 V in 0.1 V increments. **d** Drain current *I*_*d*_ in T_2_ with *V*_*x*_ ranging from 0.05 to 0.35 V, where *V*_*w*_ = 2.4 V. The bars in the right panel indicate the variation of *I*_*d*_ for each curve after T_1_ is turned off. **e** Transfer characteristics of T_2_ with *V*_*x*_ ranging from 0.05 to 0.35 V in 0.05 V increments. **f** Drain current *I*_*d*_ in T_2_ with *V*_*w*_ ranging from 2.4 to 3 V, where *V*_*x*_ = 0.1 V.

To test whether the voltage stored in the capacitor can effectively drive T_2_, we examined the time evolution of the drain current *I*_*d*_ in T_2_ after completing a storage operation. Figure 3a shows a complete diagram of the measurement circuit used to measure a 2T–1C cell’s electrical behavior, and a storage cycle is shown in Fig. 3b. The magnified area in Fig. 3b shows the storage operation in detail. *V*_*w*_ = 2.4 V with a pulse width of 140 ms, and *V*_re_ = 3 V with a pulse width of 100 ms, i.e., *V*_*w*_ rises 20 ms earlier and falls 20 ms later than *V*_re_. One should note that *I*_*d*_ has a steep pulse during a storage operation. Since it synchronizes with *V*_re_, this is mainly due to the parasitic capacitance between the gate electrode and the capacitor. After the storage operation completes and the capacitor is charged to 2.4 V, T_1_ is then turned off by applying a negative *V*_re_ (−3 V), and *V*_*w*_ is set to 0 V. Thus, the voltage potential on the capacitor entirely controls *I*_*d*_ of T_2_, without the influence of *V*_*w*_. During the 10 s holding time, the output current *I*_*d*_ decreases from 302 to 292 nA, approximately a 3% loss. It indicates that most of the charge stored in the capacitor can be maintained over a 10 s period, which keeps its voltage potential nearly constant and provides persistent control of the channel current in T_2_. Such charge storage persists even the holding time is extended to 100 s with a loss of *I*_*d*_ less than 10% (Fig. S5). Reproducibility tests show that *I*_*d*_ in T_2_ remains nearly constant after more than 100 cycles (Fig. S6). Such desirable storage characteristics indicate that, upon tuning *V*_*w*_ and *V*_*x*_, different values of *I*_*d*_ in T_2_ could be obtained and maintained with an acceptable loss in 10 s, which provides various differentiable states.

To demonstrate this, we first explored the electrical characteristics of T_2_. Figure 3c shows the output characteristics with *V*_*g*_ ranging from 2.4 to 3.0 V in 0.1 V increments, where one electrical probe is added separately to apply *V*_*g*_ directly to T_2_ as *V*_*w*_ (Fig. S7a). A relatively small *V*_*x*_ is applied to obtain linear *I*_*d*_–*V*_*d*_ output characteristics. Then *V*_*w*_ is fixed at 2.4 V, and *V*_*x*_ varies from 0.05 to 0.35 V in 0.05 V increments. Figure 3d shows *I*_*d*_–*t* curves (similar to that in Fig. 3b) under different applied *V*_*x*_ values. For each *I*_*d*_–*t* curve, *V*_*x*_ is fixed to monitor the decrease of *I*_*d*_ during one cycle (~10 s) to tell if the *I*_*d*_ at each level can be distinguished without overlap with neighboring states. The right graph shows the variation in *I*_*d*_ during one cycle. We then investigated the corresponding transfer characteristic, as plotted in Fig. 3e. *V*_*x*_ is fixed from 0.05 to 0.35 V in 0.05 V increments while *V*_*g*_ varies from 2.4 to 3 V, in which range the *I*_*d*_–*V*_*g*_ curves are all nearly linear. Figure 3f again shows the measured *I*_*d*_–*t* curves in which *V*_*x*_ is fixed at 0.1 V, and *V*_*w*_ pulse varies from 2.4 to 3 V in 0.1 V increments. Like the results in Fig. 3d, the *I*_*d*_ at each level can be distinguished in one cycle. In Fig. 3d, f, it is noteworthy that there remain charges on the capacitor at the beginning time due to the previous cycle’s operation, so that each *I*_*d*_–*t* curve has an initial value equals to that after 10 s retention time.

**Fig. 4:**
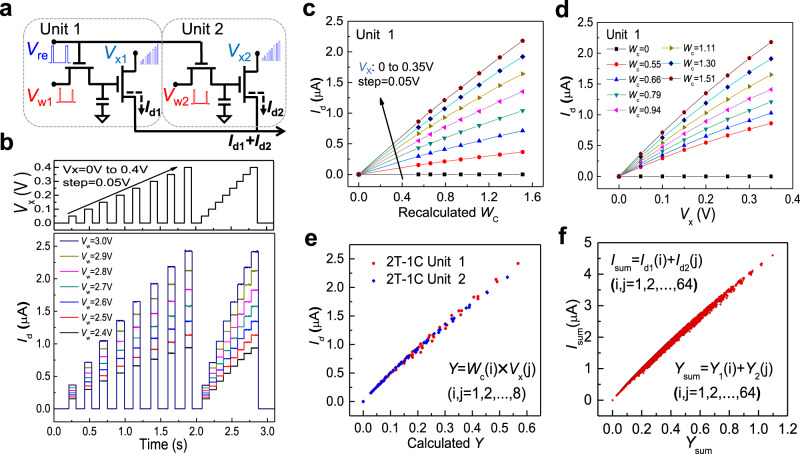
Demonstration of multiply accumulate operation using two 2T–1C cells. **a** Schematic showing two identical 2T-1C cells. The sources of the two cells are connected to sum the drain current. **b** Top graph: a test multi-step voltage waveform applied to *V*_*x*_, ranging from 0.005 to 0.35 V with 0.05 V increments. The pulse width is 0.1 s. Bottom graph: The corresponding *I*_*d*_ waveform, while *V*_*w*_ is fixed at a series of values. Both *V*_*x*_ and *V*_*w*_ exhibit eight distinguishable voltage levels (3 bits). **c** The output current *I*_*d*_ as a function of *W*_*c*_, and the fixed input *V*_*x*_ ranges from 0 to 0.35 V with 0.05 V increments. **d** The output current *I*_*d*_ is plotted as a function of *V*_*x*_ under different *W*_*c*_ values. **e** The measured *I*_*d*_ values as a function of their calculated *Y*(*W*_*c*_ × *V*_*x*_) for two different 2T–1C cells on one MoS_2_ wafer. **f** The total output current *I*_sum_ as a function of *Y*_sum_ from the two different 2T–1C cells.

As illustrated in Fig. 4a, we used two nearly identical 2T–1C cells to demonstrate a simple MAC operation. The sources of the two T_2_ cells are connected to sum up *I*_*d*1_ and *I*_*d*2_. Figure 4b shows that when a test step-waveform is applied to *V*_*x*_, and *V*_*w*_ is set as various values, *I*_*d*_ from T_2_ can be accurately controlled. *V*_*x*_ ranges from 0.05 to 0.35 V in 0.05 V increments during every test cycle, and the weighted voltage *V*_*w*_ ranges from 2.4 to 3.0 V in 0.1 V increments. The waveform *V*_*x*_ exhibits eight voltage levels (3 bits) with a pulse width of 0.1 s, while *V*_*w*_ also exhibits eight levels, spanning 7 voltage levels plus a zero level. This measurement imitates when *V*_*w*_ is stored in the 1T–1C unit, a series of operations can be performed to *V*_*x*_ to accomplish multiple calculations in a storage period. The overall speed depends on the response speed of T_2_ and the writing speed of T_1_. One should note that the output *I*_*d*_ changes almost simultaneously with the input *V*_*x*_, indicating a fast operation speed. The calculation speed depends on the response speed of the transistor T_2_, which is mainly determined by the cut-off frequency ${f}_{{\rm{T}}}=\frac{{g}_{{\rm{m}}}}{2\pi {C}_{{\rm{G}}}}$, where ${g}_{{\rm{m}}}$ is the transconductance, ${C}_{{\rm{G}}}$ is the equivalent gate capacitance^41^. Thus the upper limit of ${f}_{{\rm{T}}}$ approximately equals 127.47 kHz for our current transistor scale (details see Fig. S8), which can act as a reference value for the calculation speed. It is much lower than previously reported MoS_2_ RF devices^42,43^, mainly because the ${C}_{{\rm{G}}}$ is significantly influenced by the device size and overlap region of the gate electrode. Thus the speed improvement has a large room through fabrication optimization and further down-scaling.

We have demonstrated storage and calculation capabilities with our 2T–1C cell. We now demonstrate how to implement a MAC operation in detail. Based on the above electrical characterization of a MoS_2_ FET, we can obtain linear *I*_*d*_–*V*_*x*_ curves at small *V*_*x*_, which approach zero when *V*_*x*_ = 0. To realize the multiplication function between *I*_*d*_ and the production of *V*_*w*_ and *V*_*x*_, a linear correlation between *I*_*d*_ and *V*_*w*_ is also anticipated, i.e., a linear transfer characteristic. However, similar to previous literature results^44–46^, *I*_*d*_ has a quadratic dependence on *V*_*w*_, despite under a relatively low drain voltage regime. To achieve the required linearity, we can propose a recalculated weight

1 $${W}_{c}={({V}_{w}-1.9)}^{2}+0.3$$

Now, *I*_*d*_ and the product of *W*_*c*_ and *V*_*x*_ can fulfill the requirements of multiplication operation, i.e., ${I}_{{\rm{d}}}=\bar{k}{W}_{{\rm{c}}}{V}_{{\rm{x}}}$. The conversion between *W*_*c*_ and *V*_*w*_ can be realized by an additional peripheral circuit design (Fig. S9a). Figure 4c shows the output current *I*_*d*_ as a function of *W*_*c*_, where data was extracted from Fig. 4b, and the fixed input *V*_*x*_ ranges from 0 to 0.35 V in 0.05 V increments. For each *V*_*x*_ value, the output current *I*_*d*_ and the recalculated *W*_*c*_ show satisfying linearity. We then further investigated the relationship between *V*_*x*_ and the output current *I*_*d*_ for different *W*_*c*_ values. As shown in Fig. 4d, *I*_*d*_ is plotted as a function of eight *V*_*x*_ values with different *W*_*c*_ values. For each *W*_*c*_ value, the output current *I*_*d*_ and *V*_*x*_ are also relatively linear. Similar electrical characteristics for the second 2T-1C cell are shown in Fig. S9b, c. In the future, more linear transfer characteristics can be investigated by surface treatment and contact engineering of MoS_2_ FETs, or using gapless graphene as an alternative channel material for T_2_. So the additional peripheral circuit for linearity conversion can be simplified or removed to realize MAC operation more efficiently.

When we multiply each *W*_*c*_ (3-bit) with each *V*_*x*_ (3-bit), we obtain the mathematical product *Y* with 64 different values

2 $$Y={W}_{c}(i)\,\times\, {V}_{x}(j)\cdot (i,j=1,2,\ldots,8)$$

Figure 4e shows the measured *I*_*d*_ values of the two 2T–1C cells as a function of their corresponding *Y* values separately. *I*_*d*_ is relatively linear with *Y* for both cells. We then accumulate *Y*_1_ (cell 1) and *Y*_2_ (cell 2), defined as *Y*_sum_ = *Y*_1_(*i*) + *Y*_2_(*j*) (*i*, *j* = 1, 2, …, 64), while the corresponding sum of the output current is defined as *I*_sum_ = *I*_*d*1_(*i*) + *I*_*d*2_(*j*) (*i*, *j* = 1, 2, …, 64). Figure 4f shows a linear relationship between *I*_sum_ and *Y*_sum_.

Thus, we have shown that MAC operations can be successfully performed based on our MoS_2_ 2T–1C units. Furthermore, during the retention period, it is enough to implement multiple MAC operations upon inputting a sequence of *V*_*x*_ on T_2_. Thus our 2T–1C MoS_2_ device can be potentially used for in-situ training that can significantly improve the recognition accuracy of neural networks^47^. Therefore, our results suggest a potential path of 2D semiconductors for future post-Moore applications.

**Fig. 5:**
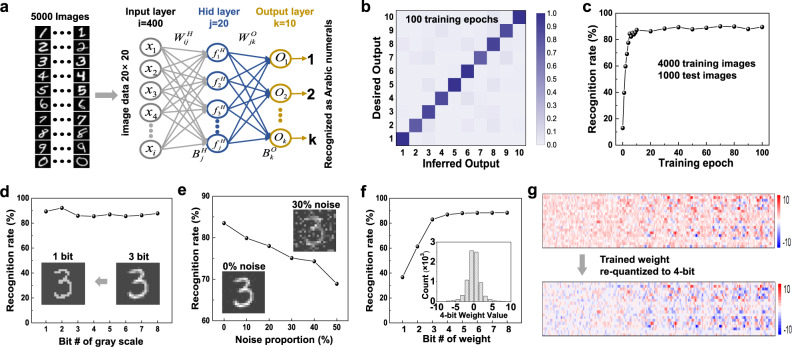
Fully connected neural network simulation for handwritten digit recognition based on experimental data. **a** Neuromorphic network with three layers, each containing 400 input neurons, 20 hidden neurons, and 10 output neurons. Where *W*_*ij*_^H^ denotes the weight between the input neuron i and the hidden neuron j, ${f}_{{\rm{j}}}^{{\rm{H}}}$ denotes the convolution value of hidden neurons j, *O*_*k*_ denotes the convolution value of the output layer, and *B*_*j*_^H^ is the bias of hidden neuron j. **b** Confusion matrix of the test results under 100 training epochs, with 4000 images used for training and 1000 images used for testing. The output 0–9 denotes the desired output handwritten digits. **c** Recognition rate as a function of training epoch (0–100), using 4000 images for training and 1000 images for testing. **d** The relationship between the recognition rate and the grayscale bit depth; the inset shows 1-bit and 3-bit grayscale input images. **e** The relationship between recognition rate and noise pixel proportion; the inset shows images with 0% noise and 30% noise. **f** The relationship between the recognition rate and quantized weight levels. The inset shows the interval distribution of 20 × 200 re-quantized 16-level weights. **g** Color map showing trained weights are re-quantized to 4-bits. The size of the colormap is 20 × 200.

Finally, we built a fully connected neural network (FNN) model with a 3-layer network for handwritten digit recognition. As shown in Fig. 5a, the 400 input neurons correspond to the 20 × 20 pixels in one image while 10 output neurons corresponded to the recognition of digits 0–9, respectively. Here, each pixel has a grayscale value from 0 and 255 (8 bits). We used 4000 images to train the simulation model and another 1000 images for testing.

To process the 8-bit grayscale data, we established an 8-bit MAC composed of 32 2T/1 C cells (Fig. S11). The trained *W*_in_ (weight of the simulation model) corresponded to quantized voltage with 256 levels (8-bit) stored in the cells. The FNN structure is shown in Fig. S12. Each 8-bit MAC works as a neuron to process the input grayscale data for each pixel. The complete FNN diagram consists of 400 × 20 neurons to form forward propagation from the input layer to a hidden layer. We used back-propagation to train our FNN simulation (see Supplementary Notes for more details). A flowchart for the training and test is shown in Fig. S13. After the FNN completed 100 training epochs against 4000 handwritten images, we performed a recognition test using 1000 handwritten images. The average recognition accuracy of our neural network simulation model reached 90.3%. Figure 5b shows the recognition confusion matrix for the 1000 images test. Figure 5c shows the relationship between recognition rate and training epoch, where the recognition rate rises quickly during the initial 10 training epochs primarily due to a large number of training images.

Considering that the size of an 8-bit grayscale input image occupies too many 2T–1C cells, we attempted to reduce the bit depth of the input grayscale images. We find that when an 8-bit input grayscale image is reduced to 1-bit, there is no evident decrease in recognition rate (Fig. 5d). We also simulated the influence of noise in our neural network by randomly choosing pixels and resetting them to random values. As shown in Fig. 5e, the in-set displays images with 0% and 30% noise levels. In the simulation, each well-trained weight is a 32-bit floating type by ex situ training, and it needs to be quantized to meet the finite weight levels. When the trained weights are re-quantized from 8 bits to 1 bit, as shown in Fig. 5f, we find that a 16-level (4-bit) weight is sufficient for our neural network to reach high recognition accuracy. The in-set in Fig. 5f shows the interval distribution of the 20 × 200 quantized 16-level weights (the quantized 256-level weights are shown in Fig. S14). Figure 5g shows a color map of the trained weights after being quantized to 16-levels. The size of the colormap is $20\times 200$. These results suggest that two 2T–1C cells are enough for a neuron to store a 4-bit quantized weight.

In conclusion, we experimentally demonstrated an in-memory computing architecture that integrates MoS_2_ FETs in a 2T–1C configuration for MAC operations. Owing to the large current on-off ratio of MoS_2_ FETs, the charge stored on the capacitor leaks slowly to present a long retention time so that a multi-level voltage can be retained. Based on the electrical characteristics of MoS_2_ FETs and an additional peripheral circuit, the analog multiplication operation can be realized with a re-calculated weight parameter. By connecting two or more 2T–1C unit cells in parallel, the output current is summed to provide the accumulation portion of a MAC operation. In addition, a neural network model was built based on the experimental data to provide image recognition with an average 90.3% accuracy. Our MoS2 2T–1C circuit is still a prototype device at the current research stage, and its performance requires further improvement by optimizing material quality and fabrication. Nevertheless, our demonstrated results offer a promising research platform for in-memory computation and in situ training of neural networks.

## Methods

### Fabrication of MoS_2_ 2T–1C cell arrays

Device fabrication begins by using photolithography (Microwriter ML3) to pattern the source/drain region and bottom capacitor plate on a monolayer MoS_2_ film grown on a sapphire substrate. The channel width/length of T_1_ and T_2_ are defined as 30/20 and 90/20 μm using ICP etching, respectively. Next, a seed layer (3 nm SiO_2_) was evaporated on the MoS_2_ film using electron beam evaporation, followed by annealing (200 °C, 10 min) in a high vacuum furnace to remove any resist residue and ensure low contact resistance. A 20-nm-thick HfO_2_ layer was then deposited using atomic layer deposition at 180 °C. The oxide stack containing 3 nm SiO_2_ and 20 nm HfO_2_ serves as a high-*k* gate dielectric of MoS_2_ FETs and the capacitor’s insulating layer as well. CF_4_/Ar plasma etching was used to form an interconnect opening in the dielectric layer to connect the source in T_1_ to the gate in T_2_. Finally, 30 nm Au was deposited as gate electrodes of the MoS_2_ FETs and the top plate of the capacitor.

### Characterization and electrical measurements

All measurements were gathered in an ambient environment at room temperature. For capacitor characterization, capacitance–voltage curves were measured with a Keysight E4990A Impedance Analyzer. The MoS_2_ FETs were characterized using a semiconductor parameter analyzer (Agilent B1500A). For dynamic memory and 2T–1C cell measurements, the Agilent B1500A was used for supplying voltage signal and detecting the channel current, and a waveform generator (Aligent 33260A) was also used to supply waveforms to the test circuit, while an oscilloscope (DS 1054Z) was used for capturing output signal voltage.

## Supplementary information

Supplementary Information files

## Data Availability

The datasets generated during and/or analyzed during the current study are available from the corresponding authors upon reasonable request.

## Code availability

The codes used for simulation and data plotting are available from the corresponding authors upon reasonable request.
