# Supplementary material for: An in-memory computing architecture based on two-dimensional semiconductors for multiply-accumulate operations
Source: Nat Commun. 2021 Jun 7;12:3347. doi: 10.1038/s41467-021-23719-3 (PMC8184885; doi:10.1038/s41467-021-23719-3)
Supplement: Supplementary file 1 — Supplementary Information files [file 41467_2021_23719_MOESM1_ESM.pdf]

Supplementary Information

An In-Memory Computing Architecture Based on Two-Dimensional Semiconductors for Multiply-Accumulate Operations

Yin Wang[1,a], Hongwei Tang[1,a], Yufeng Xie[1,a], Xinyu Chen[1], Shunli Ma[1], Zhengzong Sun[1], Qingqing Sun[1], Lin Chen[1], Hao Zhu[1], Jing Wan[1], Zihan Xu[2], David Wei Zhang[1], Peng Zhou[1,*], Wenzhong Bao[1,*]

[1] State Key Laboratory of ASIC and System, School of Microelectronics, Fudan University, Shanghai 200433, China

[2]Shenzhen Sixcarbon Technology, 188 Jiangshi Road, Shenzhen 518106, China

[a]These authors contributed equally to this work.

*Corresponding author. Email: pengzhou@fudan.edu.cn; baowz@fudan.edu.cn

Supplementary Notes

Neural network simulation

Figure S12 shows the training algorithm consisting of forward and back propagation. During forward propagation, 4000 handwritten digits were imported and the weights in the circuit were set randomly before training. After convolution from the input layer to the hidden layer, the output signal of hidden neurons is

$$f_j^H = \text{sigmoid}(\sum_{i=1}^{400} W_{ij}^H x_i(n) + B_j^H) \quad (1)$$

where $x_i(n)$ is the input pixel value of the n-th handwritten digit, W_{ij}^H denotes the weight between the input neuron i and the hidden neuron j, and B_j^H denotes the bias of hidden neuron j. Here, we used the sigmoid function as the activation function for normalization; a graph of the sigmoid function is shown in **Fig. S15**. The value of f_j^H was transferred to each output neuron.

During back propagation, delta weights (ΔW_{ij}^H) were calculated and transferred to update the hidden layer weights. The change in the value of the hidden layer weights is

$$\Delta W_{ij}^H = \eta \delta_j^H x_i(n) \quad (2)$$

where η is the learning rate and δ_j^H is the calculated error between the actual output and target output during training. When the feedback delta weights (ΔW_{ij}^H) were updated to the weights in the first layer, a training cycle was completed. The training operation from the hidden layer to the output layer is similar to that from the input layer to the hidden layer, as described above.

After the neural network was trained for 300 repetitions against 4000 handwritten digits images, we performed a recognition test using 1000 handwritten digits.

Supplementary Table

Table S1: Comparison of key features of various types of memories for in-memory computing. Ref [1-5] are the latest review papers

Table S1: Comparison of key features of different memories for in-memory computing[1,2,3,4,5,6]

	RRAM	PCM	STT-MRAM	SRAM	DRAM	Flash	**Our 2T-1C device**
Memory storage medium	Resistance (10^3-10^7 Ω)	Resistance (10^4-10^7 Ω)	Resistance (10^3-10^4 Ω)	Charge	Charge	Charge	Charge
Endurance	10^6-10^9 (limited cycling endurance)	10^6-10^9 (limited cycling endurance)	Unlimited	Unlimited	Unlimited	>10^5	Unlimited
Read/Write access time	1-100 ns (Longer write access times)	~10 ns	2~30 ns	<1 ns	~30 ns	~ 100 ns	~100 ns
Bit per cell	Multiple bits per cell	Multiple bits per cell	1 bit per cell	1 bit per 6-T	1 bit per 1T-1C	Multiple bits per cell	3 bit per 2T-1C
Device retention	Non-volatile	Non-volatile	Non-volatile	Volatile	Volatile	Non-volatile	Volatile
Write power	Medium	Medium	High	Medium	Medium	High	Medium (similar to DRAM)
Analog Computing power	Low	Low	Low	High	High	Medium	Low
Others	Large resistance window and continuous conductance	Large resistance windows; Limited working temperature;	Small resistance window; Low read signal; large write current; Good endurance as a non-volatile memory	Low density; Good technological maturity	Refresh needed; High density; Good technological maturity	Large write voltages; Limited cycling endurance; Cumbersome erase procedure; Good technological maturity	Refresh is still required ; 10 s retention; Flexible.
Reference	[7,8,9,10]	[11,12,13]	[14,15]	[16,17,18]	[19,20]	[21,22,23]	

Supplementary Figures

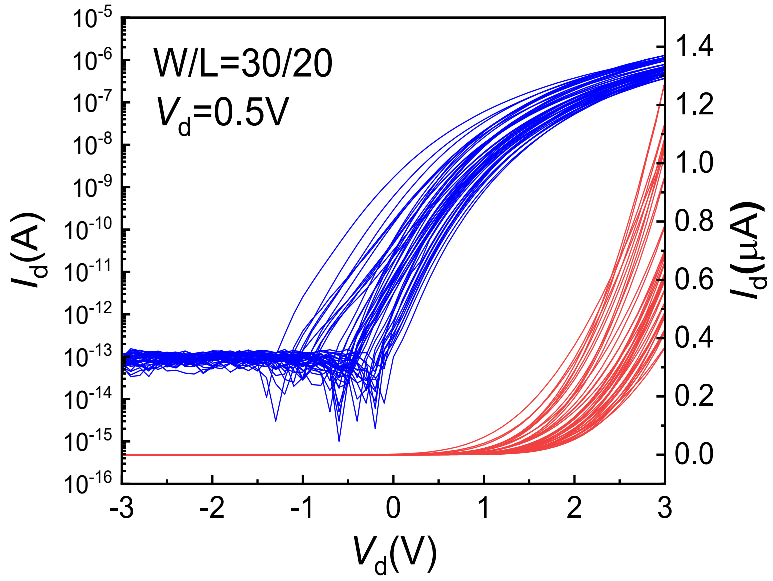

Figure S1. Twenty-four transfer curves for the MoS_2 transistors (T_1: W/L=30/20) in the 2T-1C units.

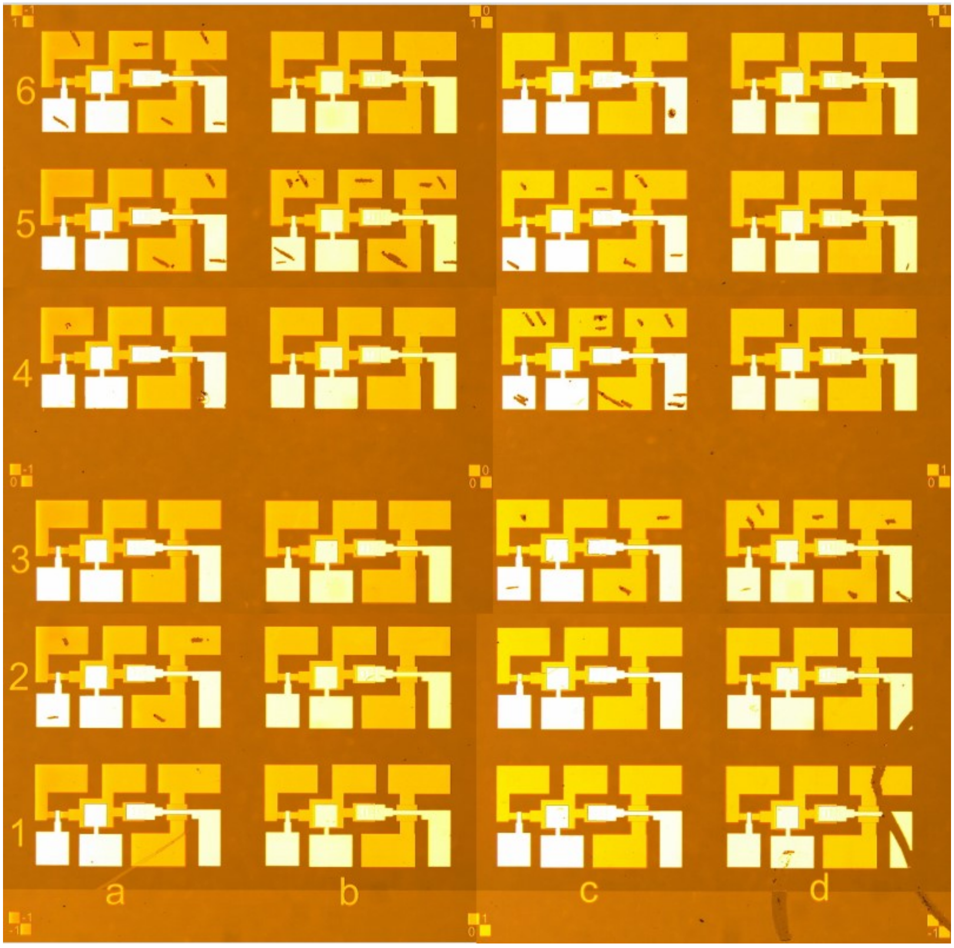

Figure S2. Microscope image of the fabricated 2T-1C unit array.

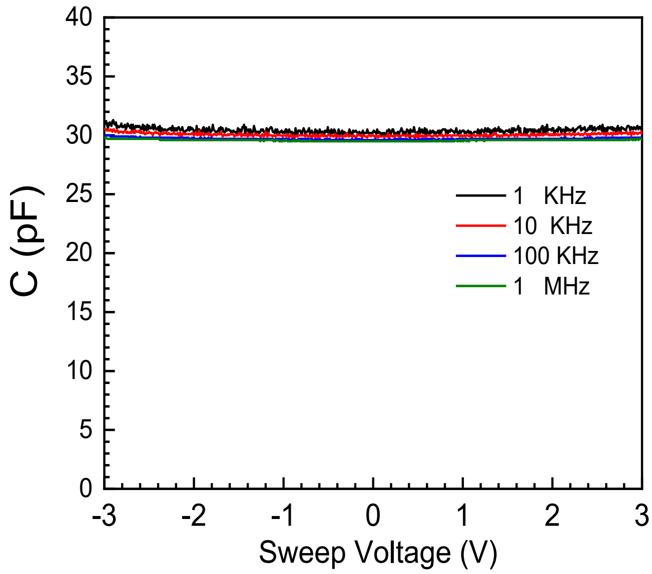

Figure S3. Capacitance-voltage curves for the capacitor with different sweep frequencies (1 kHz, 10 kHz, 100 kHz, and 1 MHz). The measured capacitance is 31 pF and the theoretical capacitance is 34.1 pF.

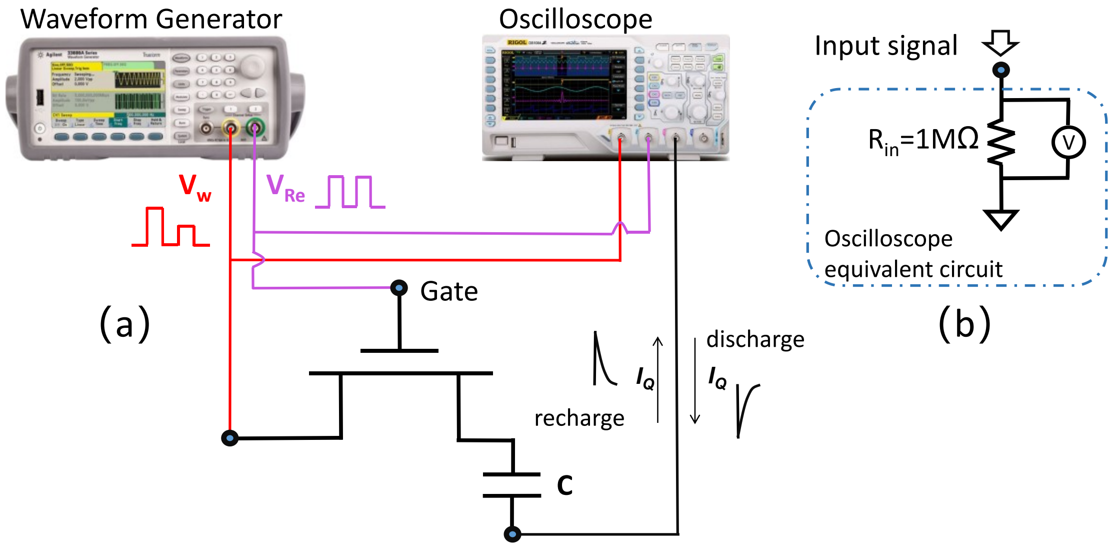

Figure S4. Schematic diagram showing electrical measurements of the 1T-1C storage module. Two multichannel pulse waveform generators were used to generate the read and write pulses with a computer program. The recharge or discharge pulse can be seen in the oscilloscope traces. The oscilloscope has 1 MΩ internal resistance.

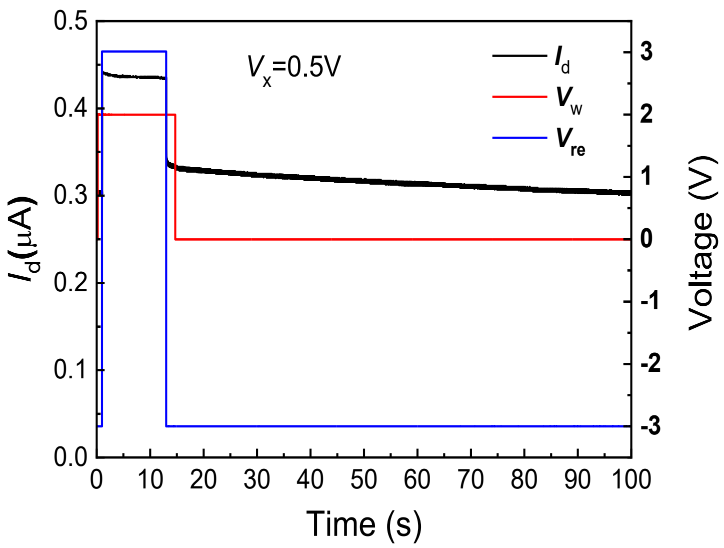

Figure S5. A complete storage and calculation operation for a 2T-1C unit when the holding time is extended to 100 s.

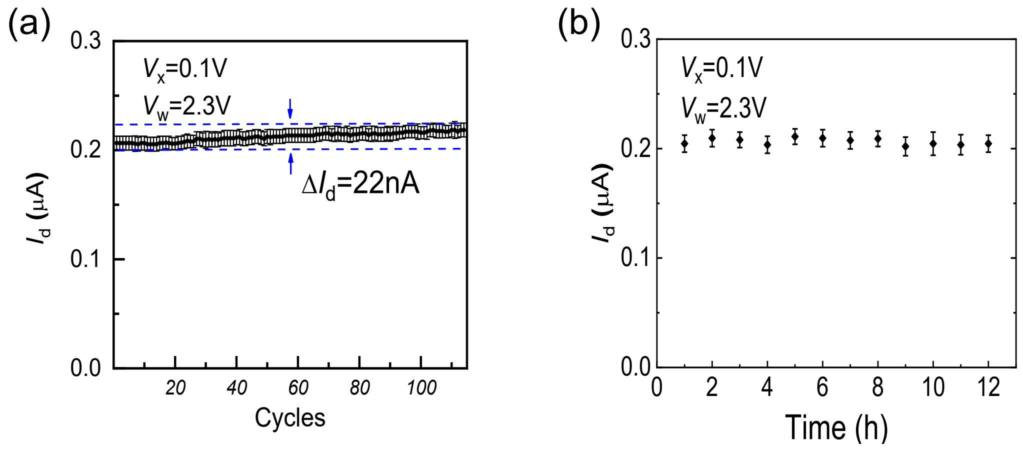

Figure S6. (a) Cycling test for a 2T-1C cell with an input voltage V_x = 0.1 V and V_w = 2.3 V. I_d varies by up to 22 nA. (b) Reproducibility test for a 2T-1C unit at 1 h intervals.

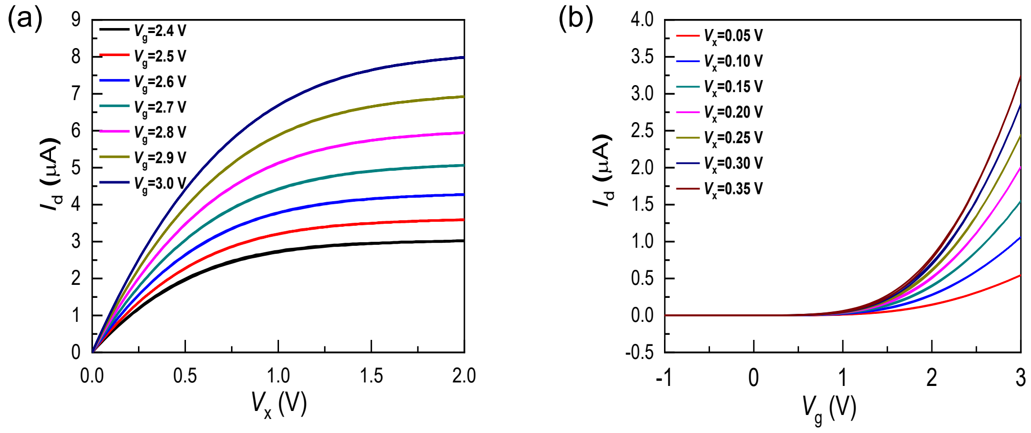

Figure S7. (a) Output from T_2 with different V_g values (one electrode is added separately to apply V_g directly to T_2 ranging from 2.4 to 3 V). (b) Transfer curves for T_2 with V_x ranging from 0.05 to 0.35 V.

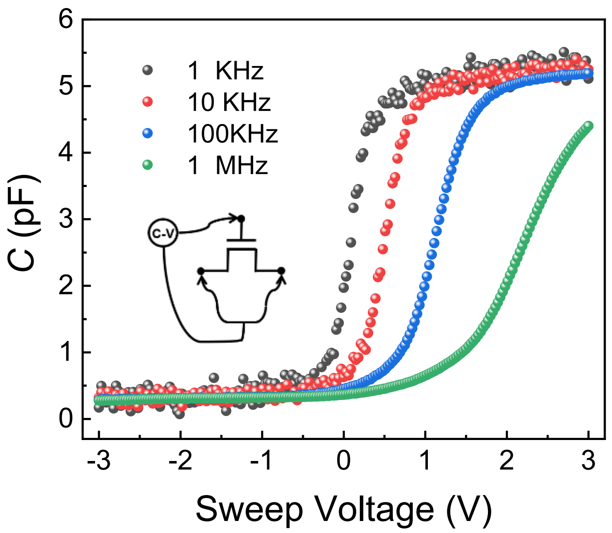

Figure S8. Capacitance-voltage curves of the MoS_2 transistor at different sweep frequencies (10 kHz, 100 kHz, 1 MHz, and 10 MHz), the insert shows the measuring schematic diagram.

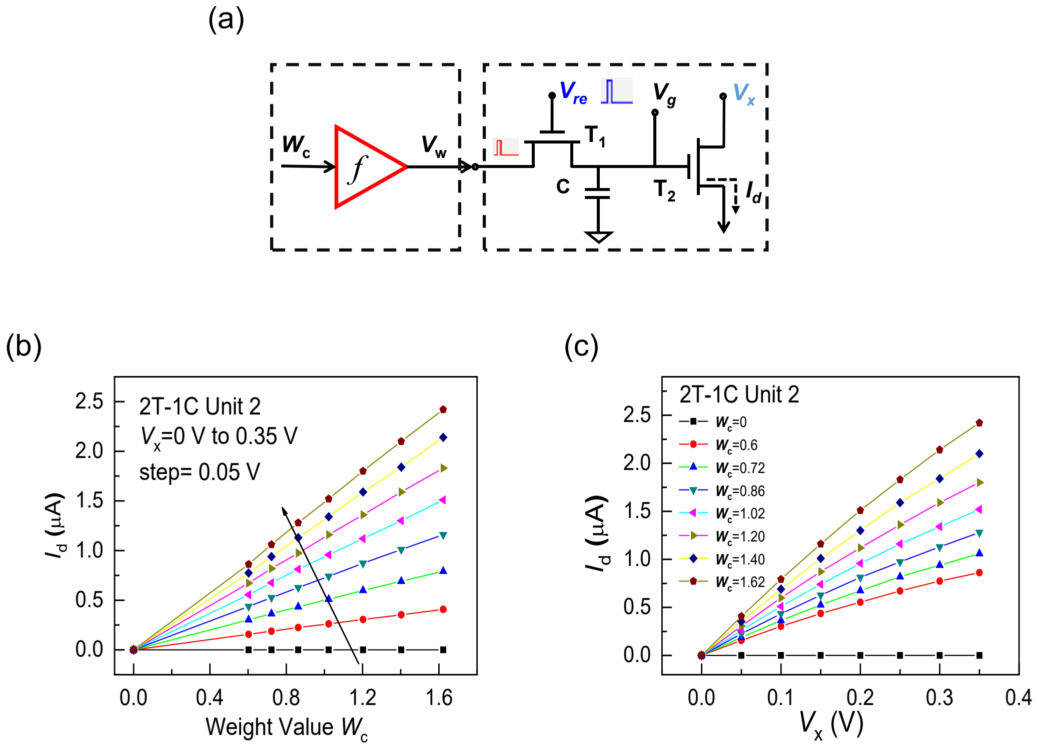

Figure S9. (a) Circuit schematic of the mapping relation f between W_c and V_w. (b) The output current I_d as a function of W_c for 2T-1C unit 2 with V_x ranging from 0 to 0.35 V. (c) The output current I_d as a function of V_x for 2T-1C unit 2 with different W_c values.

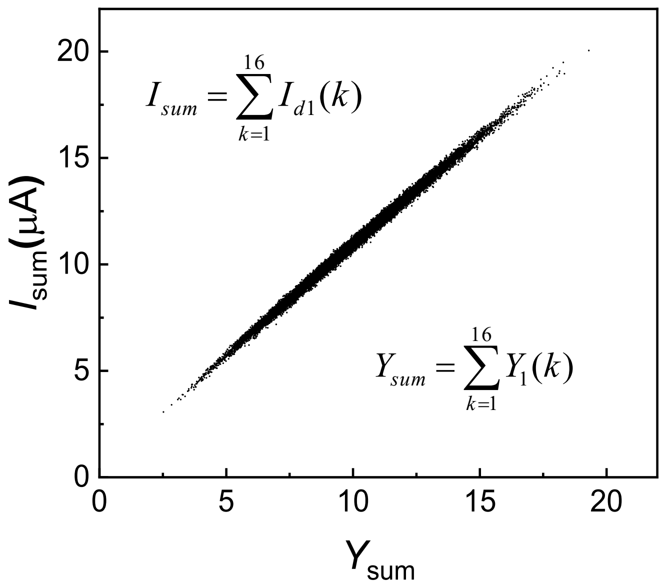

Figure S10. The simulation output of a 1×16 MAC array based on the data of 2T-1C cell 1. To further evaluate the accuracy of our MAC operation, we simulate a 4×4 MAC array based on experimental data collected from the working 2T-1C cells. By randomly generating 10000 input data, I_{sum} from the 16 cells are plotted as a function of Y_{sum} (the definition of Y_{sum} is the same as that in the manuscript), which also shows a satisfying linear relationship.

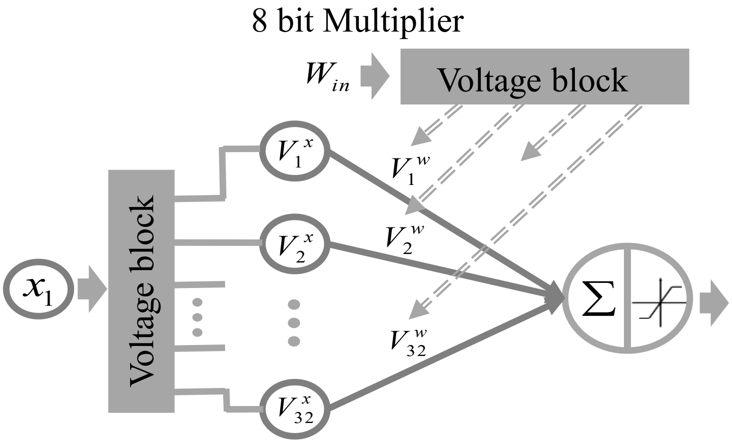

Figure S11. Network structure of an 8-bit multiplier unit with 32 cells.

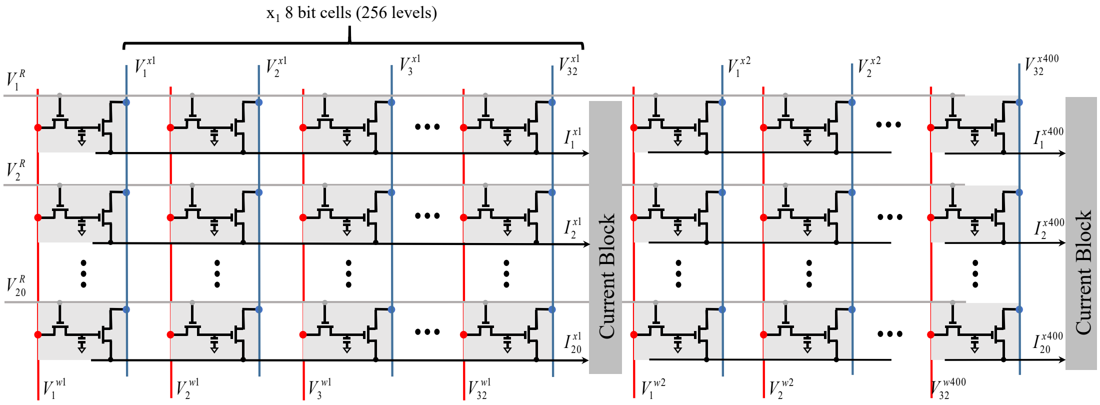

Figure S12. Diagram of a convolution circuit in a neural network structure using 2T-1C units. Each 8-bit multiplier unit works as a neuron to process 8-bit grayscale data in a single pixel.

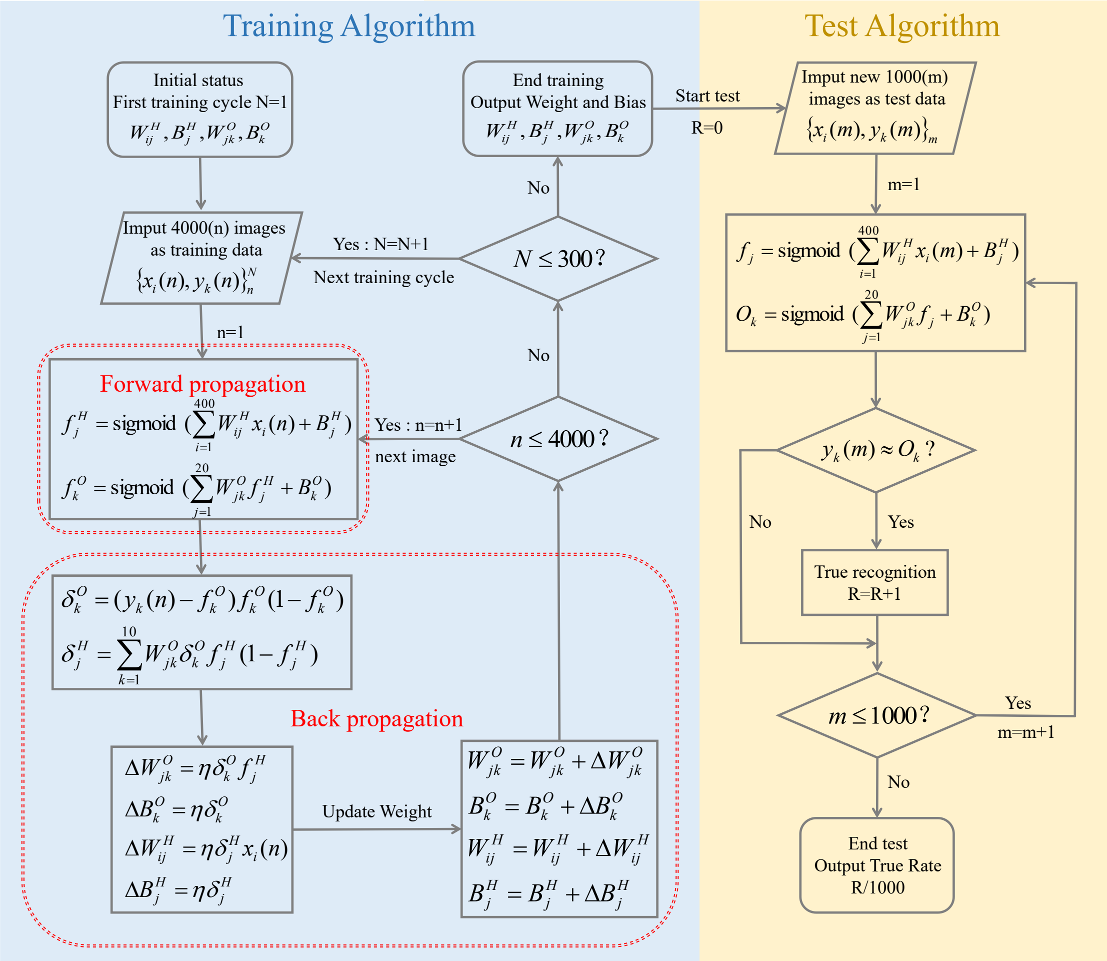

Figure S13. Training and test algorithm for the neural network, where N is the total number of training cycles and n is the total number of training images. Where W_{ij}^H denotes the weight between the input neuron i and the hidden neuron j, f_j^H denotes the convolution value of hidden neurons j, O_k denotes the output convolution value and B_j^H denotes the bias of hidden neuron j.

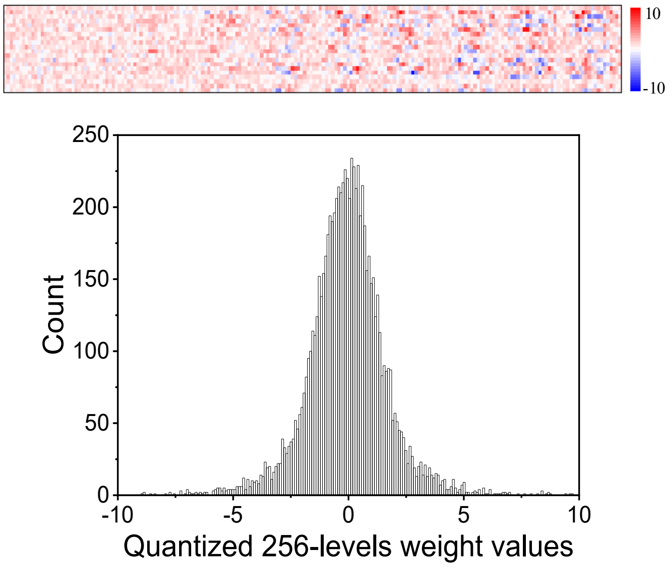

Figure S14. Distribution of the 256-level quantized weight function and its color map.

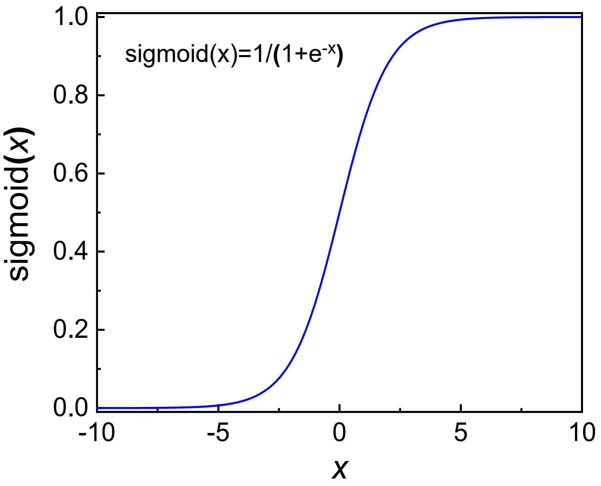

Figure S15. Graph of the sigmoid function.

References

1. Sebastian A, Le Gallo M, Khaddam-Aljameh R, Eleftheriou E. Memory devices and applications for in-memory computing. *Nat Nanotechnol* **15**, 529-544 (2020).

2. Liu C*, et al.* Two-dimensional materials for next-generation computing technologies. *Nat Nanotechnol* **15**, 545-557 (2020).

3. Wong HS, Salahuddin S. Memory leads the way to better computing. *Nat Nanotechnol* **10**, 191-194 (2015).

4. Ielmini D, Wong HSP. In-memory computing with resistive switching devices. *Nature Electronics* **1**, 333-343 (2018).

5. Xia Q, Yang JJ. Memristive crossbar arrays for brain-inspired computing. *Nature Materials* **18**, 309-323 (2019).

6. LiKamWa R, Hou Y, Gao J, Polansky M, Zhong L. RedEye: analog ConvNet image sensor architecture for continuous mobile vision. *SIGARCH Comput Archit News* **44**, 255–266 (2016).

7. Li Z, Chen P-Y, Xu H, Yu S. Design of Ternary Neural Network With 3-D Vertical RRAM Array. *IEEE Transactions on Electron Devices* **64**, 2721-2727 (2017).

8. Prezioso M, Merrikh-Bayat F, Hoskins BD, Adam GC, Likharev KK, Strukov DB. Training and operation of an integrated neuromorphic network based on metal-oxide memristors. *Nature* **521**, 61-64 (2015).

9. Cai F*, et al.* A fully integrated reprogrammable memristor–CMOS system for efficient multiply–accumulate operations. *Nature Electronics* **2**, 290-299 (2019).

10. Yao P*, et al.* Fully hardware-implemented memristor convolutional neural network. *Nature* **577**, 641-646 (2020).

11. Wang L, Gao W, Yu L, Wu J-Z, Xiong B-S. Multiple-matrix vector multiplication with crossbar phase-change memory. *Applied Physics Express* **12**, (2019).

12. Oh S, Shi Y, Liu X, Song J, Kuzum D. Drift-Enhanced Unsupervised Learning of Handwritten Digits in Spiking Neural Network With PCM Synapses. *IEEE Electron Device Letters* **39**, 1768-1771 (2018).

13. Bichler O, Suri M, Querlioz D, Vuillaume D, DeSalvo B, Gamrat C. Visual Pattern Extraction Using Energy-Efficient "2-PCM Synapse" Neuromorphic Architecture. *IEEE Transactions on Electron Devices* **59**, 2206-2214 (2012).

14. Pan Y*, et al.* A Multilevel Cell STT-MRAM-Based Computing In-Memory Accelerator for Binary Convolutional Neural Network. *IEEE Transactions on Magnetics* **54**, 1-5 (2018).

15. Kent AD, Worledge DC. A new spin on magnetic memories. *Nat Nanotechnol* **10**, 187-191 (2015).

16. Zhang J, Wang Z, Verma N. In-Memory Computation of a Machine-Learning Classifier in a Standard 6T SRAM Array. *IEEE Journal of Solid-State Circuits* **52**, 915-924 (2017).

17. Yin S, Jiang Z, Seo J-S, Seok M. XNOR-SRAM: In-Memory Computing SRAM Macro for Binary/Ternary Deep Neural Networks. *IEEE Journal of Solid-State Circuits*, 1-11 (2020).

18. Jiang Z, Yin S, Seok M, Seo J. XNOR-SRAM: In-Memory Computing SRAM Macro for Binary/Ternary Deep Neural Networks. In: *2018 IEEE Symposium on VLSI Technology*) (2018).

19. Koppula S*, et al.* EDEN: Enabling Energy-Efficient, High-Performance Deep Neural Network Inference Using Approximate DRAM. In: *Proceedings of the 52nd Annual IEEE/ACM International Symposium on Microarchitecture*). Association for Computing Machinery (2019).

20. Li S, Niu D, Malladi KT, Zheng H, Brennan B, Xie Y. DRISA: A DRAM-based Reconfigurable In-Situ Accelerator. In: *Proceedings of the 50th Annual IEEE/ACM International Symposium on Microarchitecture - MICRO-50 '17*) (2017).

21. Guo X*, et al.* Fast, energy-efficient, robust, and reproducible mixed-signal neuromorphic classifier based on embedded NOR flash memory technology. In: *2017 IEEE International Electron Devices Meeting (IEDM)*) (2017).

22. Lin Y-Y*, et al.* A Novel Voltage-Accumulation Vector-Matrix Multiplication Architecture Using Resistor-shunted Floating Gate Flash Memory Device for Low-power and High-density Neural Network Applications., 2.4.1-2.4.4. (2018).

23. Wang P*, et al.* Three-Dimensional nand Flash for Vector–Matrix Multiplication. *IEEE Transactions on Very Large Scale Integration (VLSI) Systems* **27**, 988-991 (2019).
